# Circulating Flow–Electric-Field-Configuration-Enhanced Cadmium Cementation from Sulfate Systems and Its Optimization Mechanism

**DOI:** 10.3390/ma16155463

**Published:** 2023-08-04

**Authors:** Wenjie Ding, Yunyan Wang, Weizhi Zeng, Zhumei Sun

**Affiliations:** 1 School of Metallurgy and Environment, Central South University, Changsha 410017, China; 2 National Engineering Center for Heavy Metal Pollution Control, Central South University, Changsha 410017, China; 3 School of Environmental and Safety Engineering, North University of China, Taiyuan 030051, China

**Keywords:** flow field, multi-field coupling, cementation, cadmium, kinetic

## Abstract

In this work, a novel flow–electric field coupling configuration was designed and implemented for enhancing Zn-Cd cementation. A series of tests was conducted to explore the optimization of the Zn-Cd cementation process and its mechanism. Firstly, the various characteristics of the sponge cadmium at various locations in the device were compared, and it was concluded that the optimum purity of the sponge cadmium obtained from the anode was up to 94.1%. The generation and stripping of the cadmium sponge was revealed for the first time by cross-sectional electron microscopy. The four stages of the apparent reaction in the system were analyzed in relation to the pH, cadmium concentration and cadmium sponge flaking at each flow rate. It was proved that the separation of cadmium sponge mainly occurred in the third phase. Secondly, by comparing the morphology and specific surface area of the cadmium sponge at different flow rates, the optimum flow field velocity was identified as 30 mL/s. At this point, the specific surface area reached a maximum of 1.151 m^2^/g. Six flow field configurations were compared and preferred. The results demonstrated that the LCAH (Low-Cathode-Anode-High) modulation resulted in a sparser structure of the cadmium sponge, which was more easily exfoliated from the zinc anode surface by fluid impact. This was considered to be the most beneficial flow field configuration for improving the cadmium cementation rate and reducing the cost of the reaction. Moreover, the reaction steps of the system were analyzed. Its rate-limiting step was initially empirically identified as the diffusion step and proven by calculating the activation energy of 12.6 kJ/mol. It was confirmed that the diffusion process under different flow field configurations followed the first-order kinetic principle. In addition, the system’s reaction phases were compared. Calculations confirmed that the diffusion process under various flow field configurations followed first-order kinetics. The diffusion coefficient of LACH proved to be the highest in the comparative tests, and the evident experimental results supported this conclusion.

## 1. Introduction

With the rapid growth of the battery industry and photovoltaic industry, the global production of cadmium is up to 24,000 tons [1]. Nevertheless, this trend has been limited in recent years by increasingly stringent policies in some countries [2,3]. This is mainly due to the fact that cadmium is a biotoxic heavy metal, and the treatment of cadmium pollution in nature involves huge costs and significant side effects [4,5,6]. Therefore, the production and use of cadmium in various countries is increasingly restricted [7]. Currently, cadmium products are mainly recovered in the zinc smelting industry after enrichment and refining due to their less naturally independent mineralization [8]. Thus, as one of the primary sources of cadmium pollution, the cleanliness of the zinc smelting process [9] becomes a crucial step in the clean production of cadmium.

The current research on clean and efficient cadmium extraction has focused on improving the cementation process using zinc powder, which can be more conducive to industrial applications due to its lower cost and proven process technology. Some of these investigations have optimized zinc powders’ particle size [10] and morphology [11] to improve cementation efficiency. These methods are effective in improving the Zn-Cd encapsulation problem. However, the control of Zn powder particle size adds cost. Another part of these studies attempted to optimize the reaction conditions of the zinc powder by, for example, performing graded consolidation [12] or using additives [13,14]. These studies showed that zinc powder can be reacted as wholly as possible to avoid waste. Nevertheless, using additives leads to difficulties in subsequent purification, contrary to the principles of clean smelting.

Notably, some studies have used external physical fields to optimize the Zn-Cd cementation process with good results. YANG et al. [15] proposed an electrically enhanced cadmium cementation technique that optimizes the efficiency of cadmium extraction and effectively avoids the encapsulation of the cadmium sponge on the surface of zinc powder particles. In the industrial application of electrically enhanced cadmium cementation, “floating sponge cadmium” can be quickly produced. Repeated tests have demonstrated that large amounts of “floating sponge cadmium” will cover and even connect the anode and cathode plates, resulting in the short-circuiting of the electrodes. In addition, the “floating sponge cadmium” is easily oxidized and forms dense cadmium clusters, which reduces the extraction efficiency of cadmium [16]. Based on the above, Nan et al. [17] used an ultrasonic field coupled with an existing physical field to solve the problem of floating cadmium sponges. They explored the electrochemical mechanism of the reaction process. Their results showed that the ultrasonic field effectively avoided floating sponge cadmium formation, but their specific costs were not analyzed. Owing to the high cost of industrial ultrasound equipment, using an external ultrasonic field may effectively solve the target problem but is not a comprehensive or better option for external field coupling.

In light of the above discussion, it is evident that coupling extra physical fields into the zinc–cadmium cementing electric field is an effective technique for solving the zinc–cadmium mixture [18]. The flow field condition is the most cost-effective and straightforward external field condition to acquire. The flow field changes during the electrolysis process, on the one hand, allow the electrolytes to move, making the concentration and temperature distribution more uniform, which is advantageous to the progress of electrolysis; on the other hand, the coupling of the flow field may aggravate the separation of the metal zinc and cadmium sponge, forming a new cementing activity point on the zinc surface to improve current efficiency further. Thus, maintaining the stability of the flow field within the electrolytic cell may have a beneficial effect on current efficiency enhancement. On this basis, the primary objective of this article is to construct a system that provides a circulating flow field that couples the zinc–cadmium bonding electric field to optimize the sponge cadmium purity and promote sponge cadmium settlement. Second, the zinc cadmium cementation process is investigated in a circulating flow–electric field, considering the effect of various flow field velocities on the sponge cadmium shape. Finally, the kinetic parameters were compared for various cycle configurations, and the primary mechanism for optimizing sponge cadmium was elucidated.

## 2. Experiment

### 2.1. Material

All electrodes used in the test were designed with dimensions of 40 mm × 50 mm × 3 mm, where the anode was a zinc plate (97.5% purity) and the cathode was a titanium plate (99% purity) supplied by Hunan Hongjie Chemicals Technology Co., Changsha, China. and this electrode was thoroughly cleaned and polished prior to use. The zinc electrode plate and the titanium plate were supplied by Hunan Hongjie Chemicals Technology Co., Changsha, China. All chemicals used in this study included cadmium sulfate 3/8 hydrate, zinc sulfate heptahydrate, sulfuric acid, sodium hydroxide, high-purity zinc electrode plates and high-purity titanium plates. Cadmium sulfate 3/8 hydrate, zinc sulfate heptahydrate, sulfuric acid and sodium hydroxide were all analytically pure and were supplied by Sinopharm Chemical Reagent Co., Shanghai, China. The electrolytes used in the reaction were prepared using deionized water and associated sulfates, and their specific zinc and cadmium concentrations were obtained from the samples collected from the Shuikoushan Zinc Smelter Zhuzhou Smelting Group Hunan, China. Multiple batches of samples were collected using ICP–AES. The major metal content and fractions of the reaction feedstock were determined as presented in Table 1.

Based on the original solution, a simulation solution containing cadmium solution was prepared as shown in Table 2:

### 2.2. Equipment

**Figure 1:**
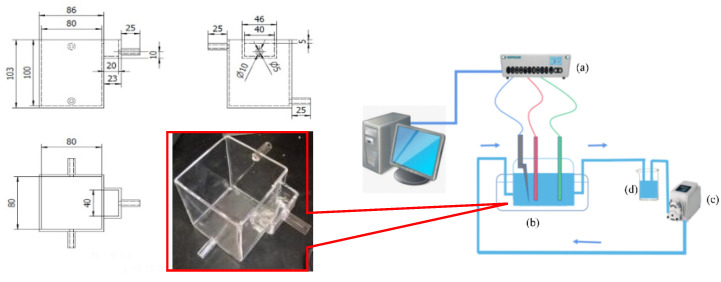
Design and assembly of a circulating flow–electric field device. (**a**) Electrochemical workstation with constant-current power source; (**b**) multi-field coupled cemented cell; (**c**) adjustable-rate peristaltic pumps; (**d**) liquid storage tank.

Figure 1 depicts the circulating flow–electric field cementing apparatus used in this work. The electrolytic cell was built from acid-resistant transparent acrylic glass with two upper and lower water inlet and exit pipes on both sides, and multiple circulation configurations were configured depending on the electrolyte flow direction. The combination of six circulating flow directions could be designed by pressing the flow direction of higherH, lowerL, cathode C, anode A, and electrode side S: H-AC-L, L-AC-H, H-CA-L, L-CA-H, LSH, and HSL. A design flow rate of around 1–30 mL/s was applied to the electrolyte circulation, which was governed by the inflow rate and total inlet area. The peristaltic pump’s input and outlet were connected to a 500 mL electrolyte storage tank, and the electrolyte was collected from the discharge outlet, mixed, and circulated.

The main experimental equipment also included the aforementioned peristaltic pump and the constant-voltage power supply module for the electrochemical workstation. Of these, the peristaltic pump (ST-HandyPump 0.03–365.69 mL/min) used in the test was supplied by innoFluid Tec Co., Shanghai, China. The Autolab electrochemical workstation (PGSTAT 302N) supplied by Metrohm China Ltd., Honkong, Switzerland. Moreover, the equipment used to pretreat the collected samples included the following: vacuum-drying oven (DZF-6050-50L) provided by Jucheng Experimental Equipment Co., Shanghai, China; reverse osmosis water purifier (1010A) by Moore Water Treatment Equipment Ltd., Chongqing, China; metallographic mounting machine (ZXQ-50S) by Laizhou Huaxing Testing Instruments Co; automatic polishing machine (Tegramin-25) supplied by Denmark Stuers Group(Shanghai), Shanghai, China.

Further, sample testing equipment included the following: inductively coupled plasma–atomic emission spectrometers ICP–AES (Angilent 5110) suplies by Angilent Technologies Co., Palo Alto, CA, USA; scanning electron microscope SEM (JSM-IT300LA) by Japan JEOL, Akishima City, Japan; X-ray diffractometer XRD (X′Pert Pro MPD) by Netherlands Malvern Panalytical Co., Shanghai, China. The specific surface area analyzer (BET Ⅱ 3020) was supplied by USA Micromeritics Tristar Co., Shanghai, China.

### 2.3. Procedure

All tests were carried out at room temperature and normal atmospheric pressure. First, 500 mL of the reaction electrolyte at the simulated concentration were poured into the reaction and storage tanks, respectively. The circulating peristaltic pump was set to 50 rpm, and the thermostatic water bath was heated to 40–80 °C and purged with nitrogen for 10 min to remove any dissolved oxygen. Finally, the distance between the electrode plates was set to 20 mm and connected to the DC power supply in constant-current mode, with the current density set to 10 mA/cm^2^. When the electrode set was inserted into the electrolytic cell, the experimental timing began.

**Figure 2:**
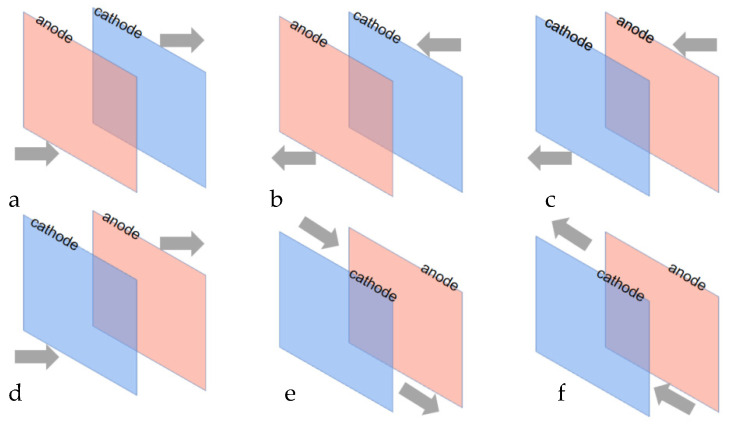
Type of flow field configuration (**a**) LACH; (**b**) HCAL; (**c**) HACL; (**d**) LCAH; (**e**) HSL; (**f**) LSH.

Based on the experimental results of the previous study [16], the optimized electric field conditions were used for the current tests to better reveal the effect of the circulating flow field on the cementation process. As shown in Figure 2, the six configurations were H-CA-L, L-CA-H, H-CA-L, L-CA-H-L, L-S-H, H-S-L; the letters H and L denote the flow ports at the high and low positions, respectively; A denotes the anode and C denotes the cathode. The order of these letters in the combination indicates the direction of flow of the fluid.

## 3. Analysis and Discussion

### 3.1. Analysis of the Apparent Reaction Process

#### 3.1.1. Cadmium Sponge Separation Process

**Figure 3:**
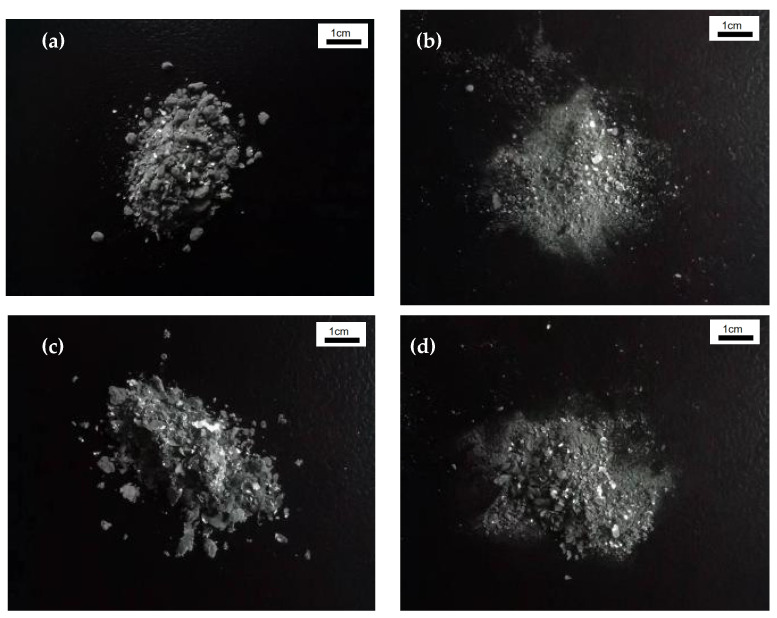
Collected sponge cadmium sample: (**a**) zinc powder sponge cadmium; (**b**) anode surface; (**c**) cell bottom; (**d**) cathode surface.

Four sets of samples were extracted to compare the morphology of the cadmium sponge. Following the linked circulating flow–electric field, three samples were recovered from the anode surface, cathode surface, and cell bottom. One batch of samples was extracted using the standard zinc powder cementation method. Surface examination revealed that all four groups of samples were greyish-white sponge metal powder (Figure 3). The presence of microscopic granular structures in the zinc powder sponge cadmium sample in Figure 3a, as well as flakes in the anode surface sample in Figure 3b and the bottom of the cell sample in Figure 3c, was the variation. Figure 3d shows that none of these structures were present in the cathode sample. These shapes represent electrode surface zinc flakes scraped off with the cadmium sponge. All four sets of samples were analyzed with ICP main components to confirm this hypothesis, and the findings are reported in Table 3.

**Figure 4:**
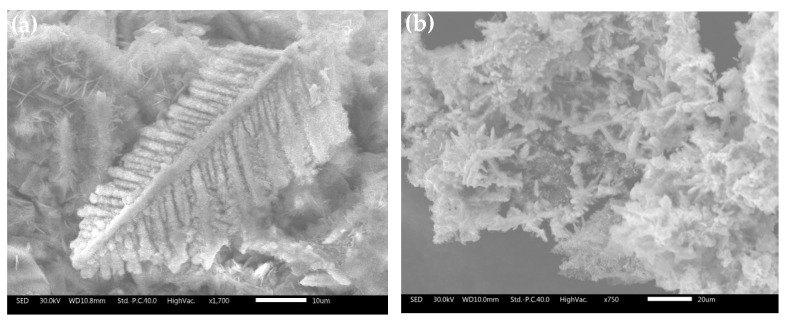

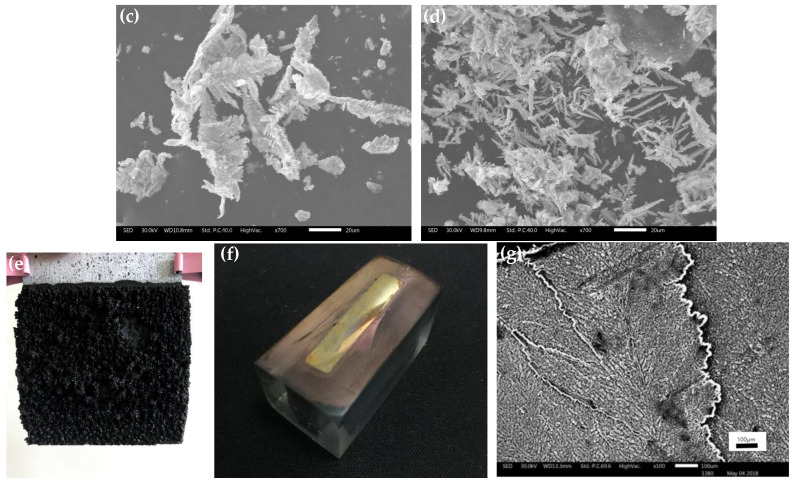
Morphology of sponge cadmium. (**a**) Dendritic structure of cadmium sponge on the anode surface; (**b**) Cadmium sponge cemented by zinc powder; (**c**) Cadmium sponge on the cathode surface; (**d**) Cadmium sponge on the anode surface; (**e**) Anode surface after reaction; (**f**) Anode section after curing and polishing; (**g**) Anode surface after cleaning of cadmium sponge under electron microscope.

Two conclusions can be drawn from the comparison of the ICP results shown in Table 3. One is that the process of using zinc powder to cement the cadmium resulted in a significantly higher zinc content in the sponge cadmium. Secondly, the composition ratios from the anode surface and cell bottom samples were very close, which can be determined because almost all of the sponge cadmium at the cell bottom was shed from the anode surface during the reaction. The samples were first collected using a filter paper funnel. They were then rinsed three times with 1 L of deionized water and further dried in a vacuum oven at 60 °C before being collected. The SEM results are shown in Figure 4.

Figure 4a shows that microscopically, the sponge cadmium consisted of feather-like dendritic structures. Compared to the conventional pellet-like sponge cadmium substituted by zinc powder Figure 4b, the sponge cadmium on the anode surface in Figure 4c and the cathode surface in Figure 4d grew in a unidirectional dendritic pattern. This resulted in less zinc being encapsulated as well as entrapped in the cadmium sponge, thus reducing the excess consumption of zinc powder. In addition to a high cadmium content of up to 95.05% Cd as shown in Table 4, both the anode and cathode also contained relatively low levels of oxygen impurities, which may have been due to the slight oxidation of the metal during sample drying. In addition, the anode sponge cadmium was significantly spongier compared to the cathode sponge cadmium, possibly related to the zinc electrolysis occurring on the anode surface. Therefore, we tried to verify this speculation by observing the growth and shedding of the cadmium sponge on the surface of the anode zinc plate.

The anode covered with the cadmium sponge was removed from the reaction device as shown in Figure 4e. After cleaning the cadmium sponge from the anode surface with deionized water, the exposed anode zinc plate was scanned by electron microscopy and the results are shown in Figure 4g. A distinct laminar structure could be observed on the surface of the anode, proving that the continuous sponge cadmium flaking from the anode was indeed due to the continuous dissolution of the laminated zinc flakes. Further, to observe the specific flaking process of the cadmium sponge and the formation of the laminated zinc flakes, the remaining anode was removed from the electrolytic cell, washed with deionized washing water, and the slices were cured in epoxy resin as shown in Figure 4f.

**Figure 5:**
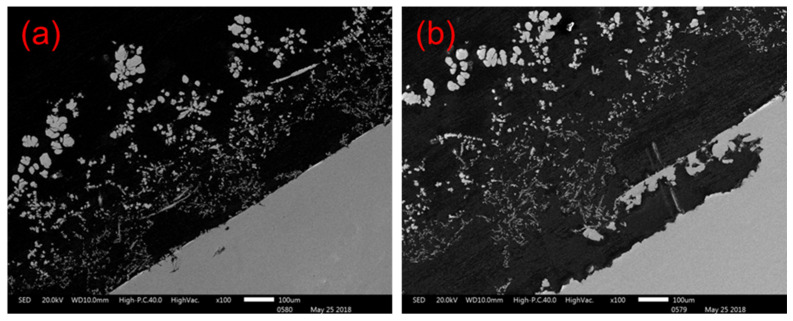
Stripping process of cadmium sponge from anode zinc plate. (**a**) Anode section for 1 h reaction; (**b**) Anode section for 2 h reaction.

Cross-sections of the anodes obtained at different times were placed under a scanning electron microscope to compare the cadmium sponge production and separation processes on the anode surface. Figure 5a shows that the cadmium sponge was extracted and attached to the anode surface. Numerous corrosion micropores appeared on the surface of the anode due to the dissolution of the zinc. Figure 5b illustrates the situation after 2 h of reaction, when the cadmium sponge grew in a dendritic pattern and gradually detached from the anode surface. The continuous dissolution and enlargement of the corrosion micropores led to the appearance of a lamellar structure and the continued exposure of unreacted zinc on the anode surface. This characterization demonstrated the sustainability of the dissolution of the zinc anode under a coupled flow field, which promoted the further detachment of the cadmium sponge from the anode.

#### 3.1.2. Apparent Reaction Process

**Figure 6:**
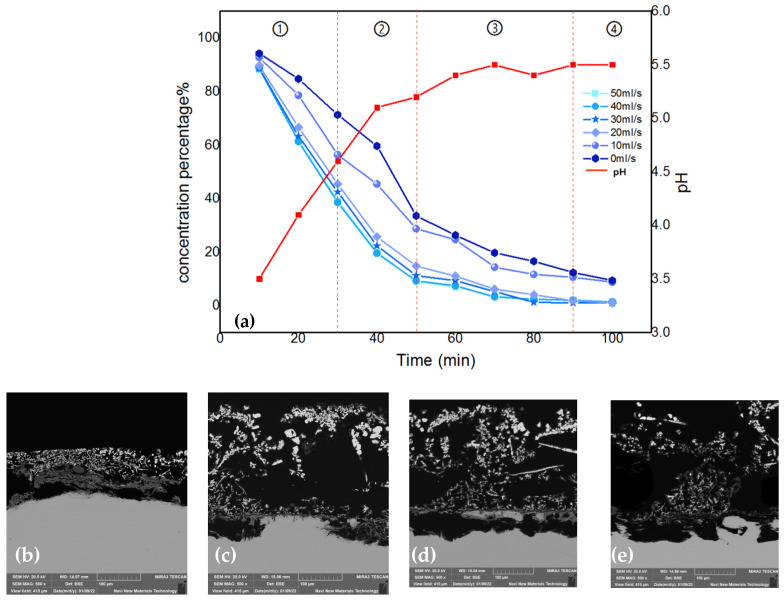
(**a**) The division of reaction phases. ① Initial reaction phase. ② Sponge cadmium formation phase. ③ Sponge exfoliation phase. ④ Reaction complete phase. (**b**) Anode morphology in first phase; (**c**) Anode morphology in second phase; (**d**) Anode morphology in third phase; (**e**) Anode morphology in last phase.

Since the above results could not visually reconstruct the separation of the cadmium sponge or other parameter changes during the cementation process, it was necessary to monitor the relevant parameters and classify the apparent reaction process based on the existing studies on optimization conditions. The flow field with the flow velocity variable coordinated as 0–50 mL/s was coupled with respect to the electric field condition with a current density of 50 mA/cm^2^ referenced from the literature [16] to facilitate the discussion of the role of the coupled flow field. The reaction parameter monitoring results are shown in Figure 6. It can be seen that the cementation process at different flow rates has four similar phases from the comparison curve of the reaction, which is named: the initial phase, the growth phase, the peeling phase, and the completion phase. The corresponding detailed judgment indexes are as follows:

(1) Initial reaction phase: The concentration decline rate is greater than (3 mg/L)/s, the pH is less than 5, and the thickness of the anode sponge cadmium is less than 3 mm.
(2) Sponge cadmium formation phase: The concentration decline rate is greater than (3 mg/L)/s, pH, and the thickness of the sponge on the anode surface is greater than 3 mm.
(3) Sponge exfoliation phase: The concentration decline rate is less than (3 mg/L)/s, the pH change is less than ±0.1, and the anode sponge cadmium is exfoliated.
(4) Reaction complete phase: The concentration percentage is close to 0, the pH change is less than ±0.1, and no new sponge appears.

As the four phases in Figure 6 show, the trend of the percentage change in cadmium concentration in the electrolyte decreased gradually over time. The coupled flow field velocity among them was zero, indicating that there was no flow field coupling in the system. Correspondingly, the percentage of cadmium concentration at the end of the reaction decreased gradually as the flow rate increased, eventually increasing by 10.37 percent when the flow rate reached its maximum. The reason for this phenomenon is thought to be that the optimized fluid conditions caused a decrease in concentration polarization at the electrode surface in this coupled multiphysics structure [19,20,21,22].

### 3.2. Moderating Effect of Circulating Flow–Electric Fields

The previous section’s conclusions revealed that the cadmium sponge’s separation from the anode occurred mainly in the third phase of the cementation reaction. Studies have shown that residual zinc in the cadmium sponge is the primary source of zinc depletion in the consolidation reaction. The dense cadmium sponge layer led to a lack of continuous zinc participation in the cementation reaction. Thus, it was necessary to identify the optimum flow field configuration to regulate the morphology of the cadmium sponge in order to investigate the influence of the mass transfer process on the crystallization and separation of the cadmium sponge and to achieve the best reaction.

#### 3.2.1. Effect of Circulating Flow Rate

**Figure 7:**
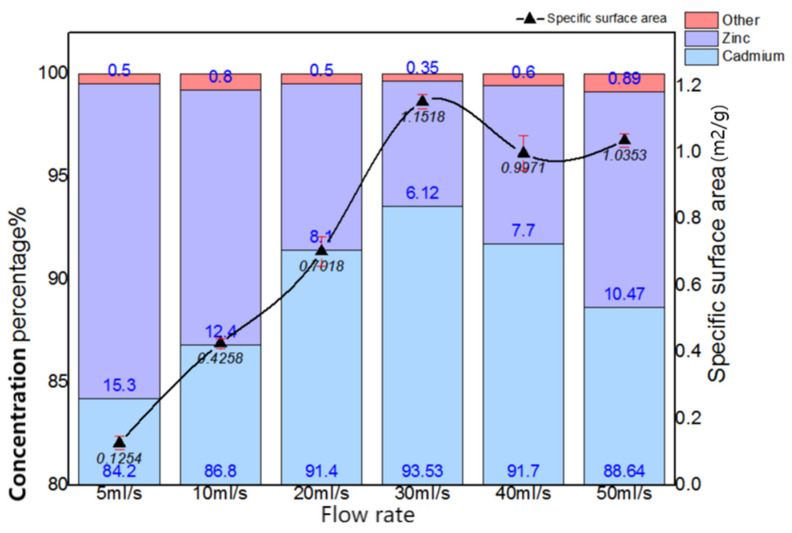
Concentration percentage (%) and specific surface area (m^2^/g) of cadmium sponge at different flow rates (mL/s).

**Figure 8:**
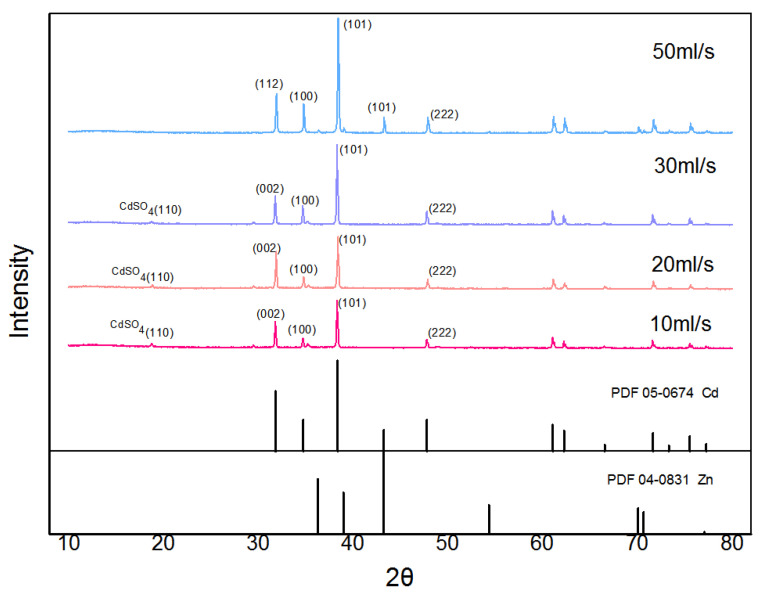
Comparison of sponge cadmium spectrum at different flow rates in XRD.

**Figure 9:**
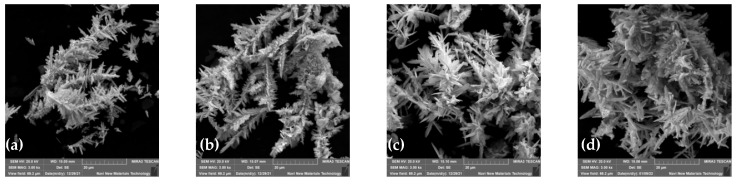
Morphology of cadmium sponge at various flow rates. (**a**) 10 mL/s; (**b**) 10 mL/s; (**c**) 40 mL/s; (**d**) 50 mL/s.

Firstly, the purity and specific surface area of the cadmium sponge at different flow rates were tested for comparison, and the results are shown in Figure 7. The purity of the cadmium sponge increased with the increase in flow rate. At a flow rate of 30 mL/s, the purity of the cadmium sponge reached a maximum of 94.93%, and then gradually decreased. Presumably, the decrease in the purity of the cadmium sponge might have been due to two factors: One is that an excessively high flow rate led to the stripping of the larger zinc on the anode surface together with the cadmium sponge into a precipitate. The other is the possible generation of alkaline zincate during the reaction. To test this speculation, the sponge cadmium was characterized by XRD and the results are shown in Figure 8. No alkaline zincates appeared in the process of cementation by comparison with the related literature studies [23,24]. Compared to other flow rates, the XRD pattern at 50 mL/s showed new peaks at 43° and 70°. This is actually one of the characteristic peaks of ZnO, which formed when the high flow rate led to the tearing of the zinc anode lamellar structure and some of the zinc entered the cadmium sponge, which was then oxidized. A small amount of incompletely washed zinc sulfate salt, on the other hand, exhibited a peak at 70°. The comparison revealed a gradual disappearance of the weak peaks near 19° 2 theta as the flow rate increased. Combined with the standard card (PDF15-0086), it is obvious that this is a characteristic peak of cadmium sulfate. The disappearance of this peak is primarily attributed to the incomplete reaction when the flow rate was slow, and the traces of residual cadmium sulfate in the cadmium sponge sample. As the flow rate increased, the reaction was more complete, resulting in no more residual cadmium sulfate in the cadmium sponge sample. Accordingly, the specific surface area of the cadmium sponge also peaked at a flow rate of 30 mL/s, as can be seen in Figure 9. This demonstrated that excessively high flow rates were not conducive to the high-purity extraction of the cadmium sponge, and that the relatively loose morphology of the cadmium sponge was more easily self-exfoliated in the presence of the flow field, resulting in less zinc stripping.

Furthermore, the cadmium sponge was collected at various flow rates and morphologically compared under electron microscopy, as shown in Figure 9. The cadmium sponge morphology showed a dense state at low flow rates and was looser at high flow rates. The reason why the flow rate affected the sponge cadmium particles is speculated as follows: when the flow rate was low, the local microscopic concentration polarization led to higher cadmium overpotential, and the nucleation rate was larger than the nucleation growth rate, so the sponge cadmium particles formed were denser [25,26]. On the other hand, when the flow rate increased, the growth rate of nucleation was higher than the rate of nucleation, resulting in the formation of looser sponge cadmium particles. The growth of the lateral branches of the cadmium sponge crystal branches were seen to be significantly stronger than the main branches as the flow rate rose [27,28,29,30]. This was presumably attributed to the growth process of the crystals being stronger than the nucleation process due to the increase in flow rate, which further demonstrated that the increase in flow rate facilitated the modulation of the spongy morphology of the cadmium sponge.

#### 3.2.2. Effect on Apparent Reaction Processes

**Figure 10:**
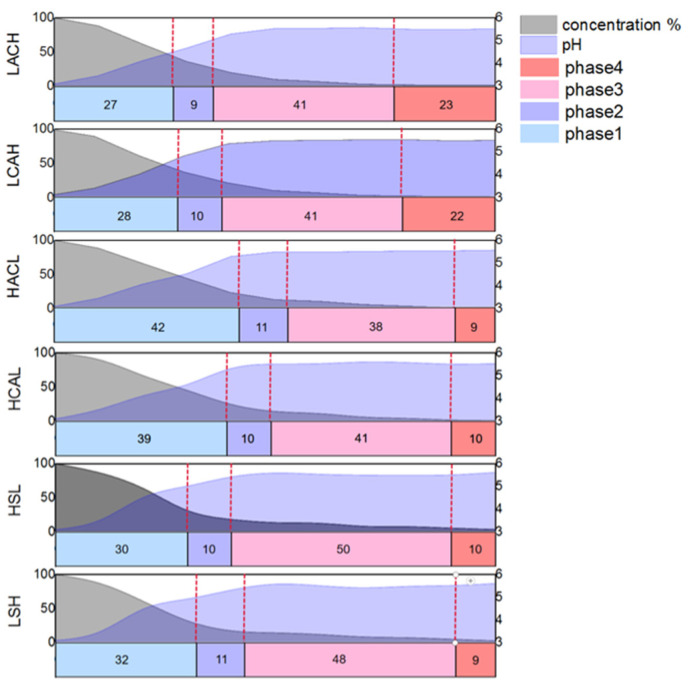
Comparison of reaction phases for different flow field configurations.

Comparative studies were performed with six distinct circulation configurations at a 30 mL/s flow rate to assess the influence of different flow field modes on the cementation process in the device. How the electrolytes are cycled affects the formation and exfoliation times of sponge cadmium. Additionally, pH and cadmium concentration variations can confirm whether the four reaction phases are accelerated or delayed. As shown in Figure 10, LACH and LCAH were the first two to enter the second phase in the six flow field configurations, owing to the turbulence in the up and down flow directions prompting earlier shedding of the cadmium sponge. For LACH, the first and second phases were completed more quickly than for LCAH, presumably due to the flow field direction from anode to cathode facilitating a greater diffusion of zinc ions and reducing concentration polarization to improve reaction efficiency. For similar reasons, the final cadmium extraction rates obtained for HSL and LSH were not improved compared to the other four configurations, but the reaction rate was still somewhat improved relative to HCAL and HACL. The reason for this was the absence of a barrier that restricted the fluid in the electrode plate. The flow direction towards the side was more conducive to the faster spread of ions in the electrolyte.

#### 3.2.3. Effect on Cadmium Sponge Morphology

**Figure 11:**
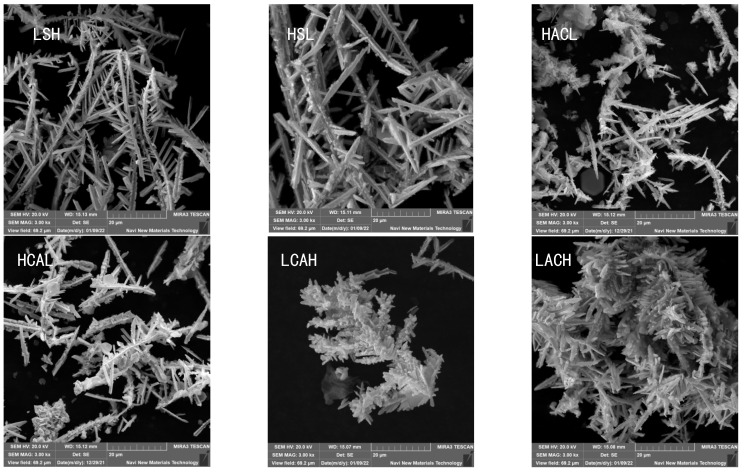
Morphology characteristics of sponge cadmium in different cyclic configurations.

**Figure 12:**
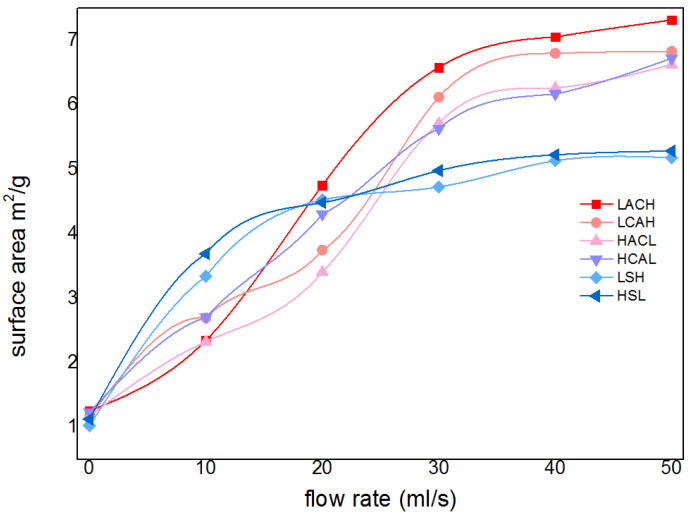
The specific surface area of sponge cadmium in different flow field configurations.

In order to confirm the findings from the previous section, we analyzed the morphology of the cadmium sponge using electron microscopy and measured its specific surface area for six different flow field configurations. These comparisons are illustrated in Figure 11 and Figure 12. It was easily found that the morphological variations of the cadmium sponges produced under different flow field configurations were mainly in terms of the dendrite development degree and specific surface area. The sample morphologies of LACH and LCAH exhibited abundant side crystal branches and dense cluster structures. When considering the information presented in Figure 12, it becomes evident that the specific surface area of LACH and LCAH exceeded that of the other four groups. Meanwhile, the specific surface area of LSH and HSL was the smallest, and their crystal branching displayed thicker main crystal branches. This confirmed the speculation in the previous section about the faster local ion diffusion in the transverse flow field.

The cadmium sponge morphology exhibited a dense state at low flow rates and was looser at high flow rates. The reason why the flow rate affected the sponge cadmium particles is speculated as follows: When the flow rate was low, the microscopic local concentration polarization led to higher cadmium overpotential, and the nucleation rate was larger than the nucleation growth rate, so the sponge cadmium particles formed were denser. Conversely, as the flow rate increased, the rate of nucleation growth was greater than the rate of crystal nucleation, resulting in looser particles of the cadmium sponge. In both configurations, LACH and LCAH, the specific surface area of the cadmium sponge was relatively large. The purity of the cadmium sponge in the other four innovative cycle configurations was essentially similar, but lower than in the two aforementioned structures. As a result of these findings, flow field conditions play a crucial role in regulating the quality of cadmium sponge, and the cyclic configuration is an effective means of optimizing and controlling the morphology of cadmium sponge.

#### 3.2.4. Effects of Element Distribution

It can be concluded from the results in the previous section that whether the cementation reaction takes place in a zinc plate or in a zinc pellet, there will be some unreacted zinc wrapped in cadmium sponge. Therefore, one of the ultimate goals of flow field modulation is to minimize the loss of zinc. Further, the ion distribution during the reaction was analyzed and compared to how the flow field configuration affected the ion distribution.

Firstly, the reactions that occurred throughout the system were analyzed. The electrolysis of zinc, the cementation of zinc and cadmium, and the electrorefining of cadmium were the three primary reactions in this system [31,32,33]. According to Faraday’s law, the mass of cadmium on cathode can be calculated from the consumption of electrical energy during the reaction process, as in Equation (1), and the specific energy efficiency ratio in the reaction process can be calculated based on the ratio of the actual collected mass of cadmium to the theoretical mass of collected cadmium, as in Equation (2).

(1) $$\frac{\mathrm{Energy}}{\mathrm{Mass}}=\frac{nFE}{M_{cd}}$$

where F is the Faraday constant with the value of *F* = 9.65 × 10,000 C/mol; n is the mol of electrons transferred in the reaction; *M_Cd_* is the molar mass of cadmium.

(2) $$\frac{m}{E}=\frac{Itmcd/nF}{UIt}=\frac{mcd}{UnF}$$

*E* refers to the electrical energy consumed; *U* refers to the voltage; *I* represents the total current applied and t represents the reaction time; *m_cd_* is the theoretical mass of collected cadmium.

Based on this, the electrical energy efficiency of each flow field configuration as well as the distribution ratio of cadmium can be calculated. The results are shown in Table 5. As shown in the table, the distribution ratio of the cadmium sponge and the corresponding electrical energy consumption changed significantly under different flow field configurations. In contrast, the anodic cementation of the cadmium sponge produced under LACH conditions was significantly higher at 75.6%. It was proved that 75.6% of its cadmium was generated by anodic cementation, and the remaining cadmium was generated at the cathode through the Faraday effect. The extent and rate of the anodic cementation reaction may be influenced by the flow field. To further substantiate this hypothesis, the reaction control process and mechanism under the reaction conditions need to be investigated.

### 3.3. Mechanistic Analysis of Flow Field Enhancement

In this section, a mechanistic study of the Zn-Cd substitution process is presented. This includes a determination of the control steps of its reaction process and an analysis regarding the kinetic processes of its reaction.

#### 3.3.1. Steps Analysis of Coupled Flow–Electric Field Cementation

**Figure 13:**
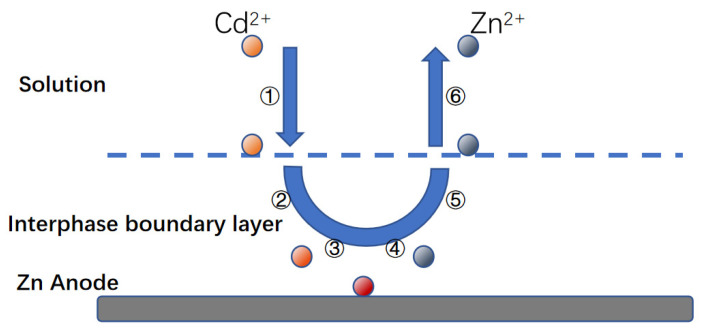
Reaction steps for Zn-Cd cementation on the anode. ① hydrated cadmium ions move to liquid phase boundary layer. ② hydrated cadmium ions across the boundary layer. ③ hydrated cadmium ions gain electrons into metal. ④ Zinc loses electrons to turn into zinc ions.⑤ Hydrated zinc ions leave the anode surface. ⑥ Hydrated zinc ions leave the boundary layer.

Zn-Cd cementation is a complex electrochemical reaction process involving multiple phases, similar to other metal substitutions in liquid. Based on our research, we believe that when combined with circulating flow–electric field coupling, the reaction process can be represented by a series of steps, as shown in Figure 13 and explained in the relevant literature [34,35,36,37]:

(1) The diffusion of hydrated cadmium ions from the solution into the around the zinc.
(2) The diffusion of these hydrated cadmium ions across the boundary layer to the cathode surface.
(3) The zinc as an anode loses electrons to the cathodic region.
(4) The hydrated cadmium ions dehydrate the film and gain electrons into metal and attached to the cathode region.
(5) Zinc loses electrons to turn into zinc ions, forming hydrated ions.
(6) Hydrated zinc ions leave the anode surface and diffuse towards the liquid phase boundary layer.
(7) Hydrated zinc ions leave the boundary layer and diffuse into the solution properly.

Typically, the first and seventh steps are not the ones that limit the process. The rate of the second and sixth steps is mostly determined by how fast the solute is transferred. The viscosity of the liquid or fluid state affects these steps, which are referred to as diffusion- or mass-transfer-limiting steps. To optimize the reaction rate of the cementation process, it’s important to identify and optimize the step that is limiting the reaction [38,39].

#### 3.3.2. Determination of Limiting Step

In the coupled circulating flow–electric field cadmium extraction process, three simultaneous reactions take place: cementation, electrorefining, and electrolysis. These reactions interact and have an impact on each other. Determining the reaction mechanism shall first determine the limiting steps of the reaction. According to the literature, the cementation reaction is typically limited by both diffusion and electrochemical factors. There are two primary ways to identify the reaction-limiting step: the empirical method and the activation energy method. The empirical method [40] is mainly based on the principle of referring to two conjugated half-reactions forming an Evans diagram with overlap and examining the location of their intersection points. The potential difference in the Tafel region is inversely proportional to the likelihood of crossover. In simplicity, the reaction-limiting step is determined by the standard electrode potential difference between the cemented metals; when this value is greater than 0.36 V for the diffusion transfer step and less than 0.06 V for the electrochemical reaction step [41]. When the value is in between, it must be determined in conjunction with other parameters. The standard potentials for Zn and Cd are −0.763 V and −0.402 V, with a potential difference of 0.361 V. This can be tentatively determined as the diffusion control [42].

It is worth noting, however, that the above potential difference is only greater than the standard value of 0.01 V for the empirical determination method. The empirical rule does not fully explain the kinetics of the cementation process as the empirical criterion Δφ0 is a thermodynamic one; the overpotential of the electrode reaction, the concentration of ions and their presence, the morphology of the surface deposits, the composition of the aqueous solution, the fluid velocity, etc. all influence the rate-limiting steps of the cementation reaction. To ensure accuracy, it is recommended to verify the results by conducting additional tests using activation energy [43].

**Figure 14:**
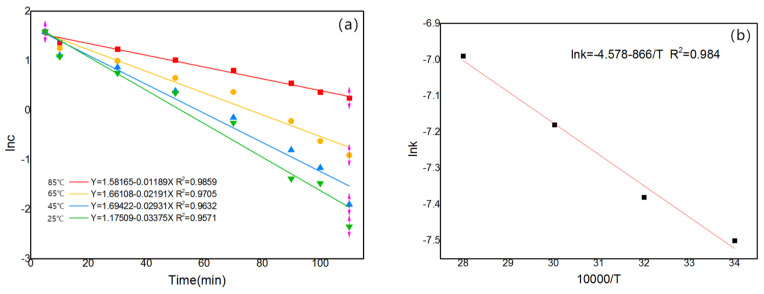
(**a**) Kinetic curve at 25 °C, 45 °C, 65 °C and 85 °C; (**b**) lnk vs. 10,000/T.

The integral method is one of the best methods to study reaction sequences and is used to evaluate first-order reactions when analyzing ln(*c*/*c*_0_) vs. time curves. Based on the concentration change curves at different temperatures (25 °C, 45 °C, 65 °C and 85 °C), the corresponding lnc vs. t curves can be obtained, as shown in Figure 14a. Further regression gives the corresponding k values. Drawing Figure 14b with lgk vs. 1/T gives a curve with a slope of E/2.303 R and an activation energy value of E. The activation energy is calculated from the slope to be 12.6 kJ/mol. The activation energy value indicates that the reaction is diffusion controlled and combined with the rule of thumb, can be determined to be diffusion controlled.

#### 3.3.3. Determination of Diffusion Coefficient

For cementation processes using geometrical electrodes, the macroscopic aspects can contribute significantly to the continuity of the reflection on the one hand, and the microscopic aspects can effectively control the complexities of the surface product on the other. For these experimental techniques, which often have very well-defined geometries and liquid flow patterns, the kinetic data can still be confusing to interpret, so relevant kinetic studies are of great importance [44].

The reaction involving cementation often results in the formation of new solids. Zinc and cadmium, however, produce cadmium sponges with a thin and loose boundary layer. This layer does not significantly affect ion diffusion kinetics. The geometry of the reactants and the state of the solid surface play a role in the reaction, and the diffusion kinetics used in this system do not align with the contraction nucleation model typically used to analyze cementation reactions [45]. Diffusion in planes mainly follows the following equations for the cementation reaction, where the plane produces a loose solid boundary layer:

(3) $$V=k(c_{1}-c_{2})=\frac{D}{\delta }(c-c_{0})$$

where *k* is the diffusion coefficient, *V* denotes the diffusion velocity, and *c* − *c*_0_ denotes the concentration difference. As shown in Equation (3), the diffusion velocity *V* is mostly determined by the diffusion constant and the concentration difference, and

(4) δ=2d(Re)−0.5=(4dηω)0.5

where *D* denotes the diffusion layer thickness and Re denotes the Reynolds number.

(5) $$D=\frac{1}{3}(\frac{RT}{\pi \eta dN})=\frac{KT}{\eta r}$$

The thickness of the diffusion layer decreases as the flow velocity increases, while the diffusion constant decreases as the temperature rises [46]. Therefore, the temperature and flow rate of a reaction are the main factors affecting the kinetic coefficient. Based on this, we can use the following formula to calculate the mass transfer coefficient for a basic batch reactor [46]:

(6) $$V_{c}\ln(c_{0}/c)=KAt$$

where *V_c_* denotes the volume of the solution and *A* denotes the effective region; *c* and *c*_0_ denote the solution’s instantaneous and starting concentrations, respectively; t denotes the reaction time.

**Figure 15:**
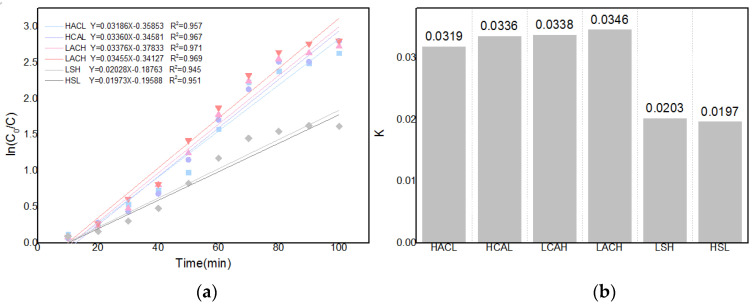
(**a**) Kinetic curves; (**b**) Mass transfer coefficients of each cycle configuration.

As illustrated in Figure 15a, linear fitting can be performed using ln(*c*_0_/*c*) and time, and slope computation can be used to determine the changing trend of the mass transfer coefficient K. As illustrated in the figure, the mass transfer coefficient K increased from 0.0137 to 0.087 as the flow rate of the circulating flow field increased from 0 to 30 mL/s. The diffusion kinetics curves for the various cyclic configurations can also be derived using the solution concentration changes, as illustrated in Figure 15. The superiority of the LACH flow field configuration is evident in its higher mass transfer coefficient, which makes it the ideal choice for facilitating the Zn-Cd cementation process. This result also provides a kinetic validation of the optimization results for each configuration in the previous section.

## 4. Conclusions

In this study, a new method of Zn-Cd gelling based on flow–electric field coupling is proposed. The cementing process, the modulation of the cadmium sponge morphology, and the associated mechanism of the technique are described in detail. The primary conclusions were obtained as follows:

Firstly, the tests compared the fractions and the morphology of sponge cadmium in this system and confirmed that the advantage of this method for sponge cadmium extraction lies in the reduction of zinc loss. Section electron microscopy was performed to reveal the initial separation process of sponge cadmium for the first time. Further, the four phases of the apparent reaction were divided according to concentration, pH and cross-sectional morphology, which provided a database for further analysis of the optimization mechanism of the method.

A series of analyses was carried out for the optimized conditions of the flow field. The reaction effect of the flow rate was tested in combination with component ratios, electron microscopy, XRD and BET data to obtain the optimum flow rate condition of 30 mL/s. The effect of each flow field configuration on the apparent reaction phases was also tested, and LACH and LCAH were confirmed to effectively end the first and second phases of the reaction earlier, thus advancing the efficient separation of cadmium sponge. Correspondingly, the cadmium sponge’s morphology and ion distribution results showed that LACH was the more suitable flow field configuration.

Analyses based on the optimization effect of this configuration were conducted regarding its reaction-limiting steps. Its reflective rate-limiting steps were calculated and determined using the empirical and activation energy methods. The results showed that the rate-limiting step of the reaction was the mass transfer step. Furthermore, the cementation reaction of zinc and cadmium in this system was shown to be a first-order reaction with an activation energy of 12.6 kJ/mol for the cementing reaction, implying that the diffusion step was the rate-limiting step, combined with a rule of thumb. After conducting more calculations using the kinetic model and performing tests on the mass transfer coefficient of different configurations, it was confirmed that LACH had a positive impact on the mass transfer optimization.

## Figures and Tables

**Table 1:** The composition of the main metal content of the solution before cadmium extraction in the zinc smelting process (mg/L).

	Zn	Cd	Fe	Cu	Co	Ni
1	50,879	22,368	406.5	338.2	4179	1175
2	63,763	18,769	317.3	419.4	6416	1650
3	47,544	26,548	195.6	455.9	4983	1774
Avg	54,062	22,561.67	306.47	404.5	5192.67	1533

**Table 2:** Concentration of original solution simulation configuration (mg/L).

Zn	Cd	Fe	Ca	Na	Mn	Mg
54,062	22,561	306.47	404.5	1493	5192.67	1533

**Table 3:** Proportion of sponge cadmium components obtained.

Location	Zinc	Cadmium
Anode	5.9%	94.1%
Cathode	12.7%	87.2%
Bottom	7.9%	92.1%
Zinc powder	26.4%	73.6%

**Table 4:** Comparison of the composition of cathodic and anodic cadmium sponges.

Location	Zinc	Cadmium
anode	4.5%	95.5%
cathode	9.3%	90.7%

**Table 5:** Comparison of ion distribution and energy consumption for different cyclic configurations.

Reaction Position	Cementation on Anode (%)	Electrorefining on Cathode (%)
No flow field	64.5	35.5
LACH	74.3	25.7
LCAH	72.5	27.5
HACL	71.6	28.4
HCAL	72.7	27.3
LSH	73.2	26.8
HSL	72.9	27.1

## Data Availability

Data is contained within the article. More data about this work are available on request from the corresponding author.

## References

[1] Callaghan R.M., Cadmium Statistics and Information|U.S Geological Survey. https://www.usgs.gov/centers/national-minerals-information-center/cadmium-statistics-and-information.

[2] Ohba K. (2018). Transport and Toxicity of Cadmium. Jpn. J. Hyg..

[3] Sandrin T.R., Chech A.M., Maier R.M. (2000). A Rhamnolipid Biosurfactant Reduces Cadmium Toxicity during Naphthalene Biodegradation. Appl. Environ. Microbiol..

[4] Renu, Agarwal M., Singh K. (2016). Heavy Metal Removal from Wastewater Using Various Adsorbents: A Review. J. Water Reuse Desalination.

[5] Ding W., Wang Y., Zeng W., Xu H., Chen B. (2023). Preparation of Heavy Metal Trapping Flocculant Polyacrylamide-Glutathione and Its Application for Cadmium Removal from Water. Polymers.

[6] Li Z., Liang Y., Hu H., Shaheen S.M., Zhong H., Tack F.M.G., Wu M., Li Y.-F., Gao Y., Rinklebe J. (2021). Speciation, Transportation, and Pathways of Cadmium in Soil-Rice Systems: A Review on the Environmental Implications and Remediation Approaches for Food Safety. Environ. Int..

[7] Okereafor U., Makhatha M., Mekuto L., Uche-Okereafor N., Sebola T., Mavumengwana V. (2020). Toxic Metal Implications on Agricultural Soils, Plants, Animals, Aquatic Life and Human Health. Int. J. Environ. Res. Public Health.

[8] Achternbosch M., Kupsch C., Sardemann G., Bräutigam K.-R. (2009). Cadmium Flows Caused by the Worldwide Production of Primary Zinc Metal. J. Ind. Ecol..

[9] Zhang C., Min X., Zhang J., Wang M., Li Y., Fei J. (2016). Reductive Clean Leaching Process of Cadmium from Hydrometallurgical Zinc Neutral Leaching Residue Using Sulfur Dioxide. J. Clean. Prod..

[10] Safarzadeh M.S., Moradkhani D., Ilkhchi M.O. (2007). Determination of the Optimum Conditions for the Cementation of Cadmium with Zinc Powder in Sulfate Medium. Chem. Eng. Process. Process Intensif..

[11] Younesi S.R., Alimadadi H., Alamdari E.K., Marashi S.P.H. (2006). Kinetic Mechanisms of Cementation of Cadmium Ions by Zinc Powder from Sulphate Solutions. Hydrometallurgy.

[12] Rao M.D., Meshram A., Verma H.R., Singh K.K., Mankhand T.R. (2020). Study to Enhance Cementation of Impurities from Zinc Leach Liquor by Modifying the Shape and Size of Zinc Dust. Hydrometallurgy.

[13] Kim M.J., Park I.J., Kim D.W., Jung H.C. (2019). A Study on the Cementation Reaction of Cadmium by Zinc Powders from Leaching Solution of Waste Nickel-Cadmium Batteries. J. Korean Inst. Resour. Recycl..

[14] Taha A., SaHa E.-G. (2004). Effect of Surfactants on the Cementation of Cadmium. J. Colloid Interface Sci..

[15] Hu Q., Yang J., Nan T., Xie X., Ye Y. (2022). Study on the Electrically Enhanced Process for Cadmium Removal by a Pulse in a Sulfuric Acid System. Process Saf. Environ. Prot..

[16] Ding W., Zeng W., Wang Y., Xu H., Chen B., Zheng X. (2022). Cadmium Depth Separation Method in Polymetallic Sulfate Solution: Flow-Electric Field Enhanced Cementation Combined with M5640 Extraction. Inorganics.

[17] Nan T., Yang J., Wang W., Li L., Yang J. (2019). Process and Anodic Reaction Mechanism of Cadmium Electrically Enhanced Cementation on Zinc Plate under an Ultrasonic Field in Ammoniacal System. Trans. Nonferrous Met. Soc. China.

[18] Ku Y., Wu M.-H., Shen Y.-S. (2002). A Study on the Cadmium Removal from Aqueous Solutions by Zinc Cementation. Sep. Sci. Technol..

[19] Aurousseau M., Pham N.T., Ozil P. (2004). Effects of Ultrasound on the Electrochemical Cementation of Cadmium by Zinc Powder. Ultrason. Sonochem..

[20] Pham N.T., Aurousseau M., Gros F., Ozil P. (2005). Improvement of a Cementation Process by Ultrasound: Case of the Cadmium? Zinc Couple at a RDE. J. Appl. Electrochem..

[21] Amin N.K., El-Ashtoukhy E.-S.Z., Abdelwahab O. (2007). Rate of Cadmium Ions Removal from Dilute Solutions by Cementation on Zinc Using a Rotating Fixed Bed Reactor. Hydrometallurgy.

[22] Zhang L., Cheng J., Yang Y., Wen Y., Wang X., Cao G. (2008). Study of Zinc Electrodes for Single Flow Zinc/Nickel Battery Application. J. Power Sources.

[23] Huang Y., Geng Y., Han G., Cao Y., Peng W., Zhu X., Zhang T., Dou Z. (2020). A Perspective of Stepwise Utilization of Hazardous Zinc Plant Purification Residue Based on Selective Alkaline Leaching of Zinc. J. Hazard. Mater..

[24] Milchev A. (2011). Electrochemical Phase Formation: Some Fundamental Concepts. J. Solid State Electrochem..

[25] Guo L., Oskam G., Radisic A., Hoffmann P., Searson P.C. (2011). Island Growth in Electrodeposition. J. Phys. D Appl. Phys..

[26] Wu Z., Yang S., Wu W. (2016). Shape Control of Inorganic Nanoparticles from Solution. Nanoscale.

[27] Cervantes R.L., Murr L.E., Arrowood R.M. (2001). Copper Nucleation and Growth during the Corrosion of Aluminum Alloy 2524 in Sodium Chloride Solutions. J. Mater. Sci..

[28] Isaev V.A., Grishenkova O.V., Zaikov Y.P. (2018). Theory of Cyclic Voltammetry for Electrochemical Nucleation and Growth. Small.

[29] Wright T.W., Ramesh K.T. (2009). Statistically Informed Dynamics of Void Growth in Rate Dependent Materials. Int. J. Impact Eng..

[30] Yang D., Xie G., Zeng G., Wang J., Li R. (2006). Mechanism of Cobalt Removal from Zinc Sulfate Solutions in the Presence of Cadmium. Hydrometallurgy.

[31] Chen J., Lei Y., Zhu C., Sun C., Xu Q., Cheng H., Zou X., Lu X. (2022). Morphology and Distribution of Cemented Product Formed via Cementation over Zn in Zinc Sulfate Solution Relevant to Roast-Leach-Electrowin Process. Hydrometallurgy.

[32] Gonçalves D.C.A., Majuste D., Ciminelli V.S.T. (2021). Improvements in the Selective Cementation of Cd and Ni/Co from Zinc Industrial Electrolyte. Hydrometallurgy.

[33] Abdel-Rahman H.H., Moustafa A.H.E.-D., Abd-Elhamid S.M., Kassem M.G.A.A. (2014). Recovery of Copper from Synthetic Solution by Cementation on Moving Bead of Zinc Spheres. Electrochemistry.

[34] Wang L., Gui W., Teo K.L., Loxton R., Yang C. (2012). Optimal Control Problems Arising in the Zinc Sulphate Electrolyte Purification Process. J. Glob. Optim..

[35] Viramontes Gamboa G., Medina Noyola M., López Valdivieso A. (2005). The Effect of Cyanide and Lead Ions on the Cementation Rate, Stoichiometry and Morphology of Silver in Cementation from Cyanide Solutions with Zinc Powder. Hydrometallurgy.

[36] Serdiuk V., Pavlenko I., Bolshanina S., Sklabinskyi V., Włodarczak S., Krupińska A., Matuszak M., Bielecki Z., Ochowiak M. (2023). Kinetic Features of Cd and Zn Cathodic Formations in the Membrane Electrolysis Process. Fluids.

[37] Liu X., Wang S., Peng Z., Zhang G., Gui Q., Zhang L. (2023). Removal of Toxic Cadmium (II) from Zinc Sulfate Solution with Zinc Powder Enhanced by Ultrasound: Kinetics and Mechanism. Sep. Purif. Technol..

[38] Korolczuk M., Stepniowska A., Tyszczuk K. (2009). Determination of Cadmium by Stripping Voltammetry at a Lead Film Electrode. Int. J. Environ. Anal. Chem..

[39] Demirkıran N., Ekmekyapar A., Künkül A., Baysar A. (2007). A Kinetic Study of Copper Cementation with Zinc in Aqueous Solutions. Int. J. Miner. Process..

[40] Wang Z., Li Y., Ye C. (2011). The Effect of Tri-Sodium Citrate on the Cementation of Gold from Ferric/Thiourea Solutions. Hydrometalurgy.

[41] Sulka G.D., Jaskuła M. (2005). Influence of the Sulphuric Acid Concentration on the Kinetics and Mechanism of Silver Ion Cementation on Copper. Hydrometallurgy.

[42] Jeon S., Bright S., Tabelin C.B., Kuze A., Ito M., Hiroyoshi N. (2022). A Kinetic Study on Enhanced Cementation of Gold Ions by Galvanic Interactions between Aluminum (Al) as an Electron Donor and Activated Carbon (AC) as an Electron Mediator in Ammonium Thiosulfate System. Minerals.

[43] Makhloufi L. (2000). Removal of Lead Ions from Acidic Aqueous Solutions by Cementation on Iron. Water Res..

[44] Aktas S. (2011). Rhodium Recovery from Rhodium-Containing Waste Rinsing Water via Cementation Using Zinc Powder. Hydrometallurgy.

[45] Farahmand F., Moradkhani D., Safarzadeh M.S., Rashchi F. (2009). Optimization and Kinetics of the Cementation of Lead with Aluminum Powder. Hydrometallurgy.

[46] Sędzimir J.A. (2002). Precipitation of Metals by Metals (Cementation)—Kinetics, Equilibria. Hydrometallurgy.

